# Nass use and associated factors among outpatients in northern Afghanistan: A cross-sectional study in Andkhoy City

**DOI:** 10.18332/tid/93574

**Published:** 2018-08-24

**Authors:** Mohammad Hasssan Hamrah, Mohammad Shoaib Hamrah, Mohammad Hussain Hamrah, Toba Dahi, Akbar Fotouhi, Junichi Sakamoto, Mohammad Hashem Hamrah

**Affiliations:** 1 Mohammad Hashem Hamrah’s Curative Clinic, Andkhoy, Afghanistan; 2 Faculty of Stomatology, Kunduz University, Kunduz, Afghanistan; 3 Department of Cardiology, Nagoya University Graduate School of Medicine, Nagoya, Japan; 4 Arya University Faculty of Medicine, Mazar-i-Sharif, Afghanistan; 5 Faculty of Dentistry, Abant Izzet Baysal University, Bolu, Turkey; 6 Department of Epidemiology and Biostatistics, School of Public Health, Tehran University of Medical Sciences, Tehran, Iran; 7 Tokai Central Hospital, Kakamigahara, Japan

**Keywords:** associated factors, Afghanistan, nass use

## Abstract

**INTRODUCTION:**

Tobacco use is one of the main risk factors for a number of chronic diseases, including cancer, lung diseases, and cardiovascular diseases. Nass is a form of smokeless tobacco. It is used most commonly in Central Asia, Afghanistan, Pakistan, and Iran, and is significantly associated with oral and oesophageal cancers. The aim of this study was to determine the prevalence of nass use and its associated factors among patients attending an outpatient clinic in Afghanistan.

**METHODS:**

This cross-sectional study was performed in an outpatient clinic in Andkhoy, Afghanistan, from April to July 2017. The study included 387 consecutive patients. The data collection tool was a questionnaire, which includes three main items: demographic, physical, and biomedical measurements. We performed binary logistic regression to identify independent risk factors of nass use.

**RESULTS:**

The study included 387 participants, of whom 225 were males (58.1%) and 162 were females (41.9%). The overall prevalence of nass use was 48.8% (95% CI: 43.8–53.9%), with the Turkmen ethnic group having significantly higher prevalence than other ethnic groups (58.2%, 95% CI: 50.8–65.3% vs 41.8%, 95% CI: 34.7–49.2%). The mean and standard deviation of the age was 63.9 (17.2) years for nass users and 42.3 (17.4) years for non-users.

Based on a multivariate analysis, family history of nass use (OR=31.4, 95% CI: 12.5–78.5), illiteracy (OR=8.9, 95% CI: 2.5–31.2), rural residence (OR=2.0, 95% CI: 1.0–3.9), and unemployment (OR=5.4, 95% CI: 1.4–21.4) were associated with nass use.

**CONCLUSIONS:**

Our results indicate that about half of the participants were nass users, and nass use associated factors were family history of nass use, illiteracy, unemployment, and rural residency in outpatient clinics in Afghanistan. More surveillance data are needed on a national level to inform the development of strategies for the prevention and control of nass consumption in Afghanistan.

## INTRODUCTION

Tobacco use is a major risk factor for many chronic diseases including cancer, lung diseases, and cardiovascular diseases^1^. Annually, tobacco use accounts for about 7 million deaths globally. More people die from tobacco use than from HIV/AIDS, malaria and tuberculosis combined^2^. The current trend shows that the number of deaths attributed to tobacco use will rise to more than 8 million annually by 2030^3^. Tobacco can either be smoked or consumed in a smokeless form. Smokeless tobacco (SLT) causes oral, head and throat cancer^4,5^.

The prevalence of smoking has been decreasing in most developed countries but increasing in low-and middle-income countries where 80% of smokers live^6^. However, the use of SLT products in many developed and developing countries has not changed^7^. Nearly 20% of the world’s tobacco is consumed in smokeless form. Its consumption is common amongst people in South Asia^8^. The use of SLT is more common amongst people in Asia, Africa and the Middle East than in western countries^9^.

SLT is one of the major risk factors associated with the high prevalence of oral, head and throat cancer in South Asia^10^. There are different types of SLT products in different parts of the world, such as traditional chewing tobacco and snuff, and new products such as compressed tobacco lozenges, tobacco chewing gum, and dissolvable strips^11^. SLT is called nass in Afghanistan, Iran, Pakistan, and in central Asian Republics. Nass is a mixture of tobacco leaves, lime, cotton oil or sesame oil, and ash^12^. Nass is used by placing it in the oral cavity under the tongue or between the oral mucosa and gingival cavity^13^. A previous study conducted in Pakistan reported that the chemical constituents of nass are linked to cancer. Nass is attributed to oral and esophageal cancers^14^. The risk of developing oral cancer is more than 20 times higher in nass users compared to nonusers^15^.

Due to three decades of conflict, there is little information available about the prevalence of nass use in Afghanistan. According to a recent study conducted in Kabul regarding the burden of hypertension, the prevalence of nass use among adults was reported to be 9.8%^16^. Verifying data on the burden and factors associated with nass use among the Afghan population might provide a better understanding of the pattern of nass use and help in developing interventions and health policies. Therefore, the aim of this study was to determine the prevalence of and factors associated with nass use among patients attending an outpatient clinic in Afghanistan.

## METHODS

### Study design

This cross-sectional study was conducted in an outpatient clinic in Andkhoy, Afghanistan, from April 2017 to July 2017. The northern part of Afghanistan was identified as a suitable place to study this smokeless tobacco product and its associated factors.

In all, 387 consecutive patients were included in the study. In brief, the case subjects were patients 18–80 years old who were physically able and willing to participate. The exclusion criteria were patients being very ill or over 80 years old. The study was approved by the scientific review committee of Balkh Regional Hospital.

### Measurements

The patients were evaluated during their clinic visit using the WHO STEPwise questionnaire^17^, which includes three main items: demographic, physical, and biochemical measurements. The questionnaire was modified by expanding on some optional questions in order to make them more relevant to the specific type of tobacco use. Nass use was included in the survey protocol. The questionnaire was translated into the Dari language and tested to check the effectiveness of the research process (the results of the pilot study was not included in the analysis).

Trained doctors collected relevant data on the nass use, demographic, and clinical characteristics of participants using the WHO STEPwise questionnaire. Demographic variables included items on age, sex, educational level (illiterate, primary/private education, secondary education, university/other higher education), marital status (single, married, other), ethnicity (Turkmen or other ethnic groups), occupation (employed, unemployed, housewife, farmer), place of residence (rural or urban), and participants’ age groups (≤ 24, 25–44 and ≥ 45 years). The study protocol included assessment of data obtained from each patient. The second part of the questionnaire included data on other factors outlined below.

### Body mass index (BMI)

The procedure for calculating the BMI (weight in kilograms divided by height in meters squared) of participants was as follows: height was measured without shoes to the nearest 0.5 cm. Before measuring weight, we asked the patients to remove heavy items from their pockets and remove heavy items of clothing or apparel (i.e. big jackets, shoes). We used an electronic scale with accuracy to the nearest 100 g. The procedure for measuring weight was as follows: the scale was zeroed before the patient stepped onto it, then asked to look straight ahead and stay still on the scale, while the researcher waited for the measurement to stabilize before recording it.

The definition of BMI as a measure of obesity varies across studies, and even within the same country. For this study, overweight was defined^18^ by a BMI of 25 to <30 kg/m^2^ and obese by a BMI ≥30 kg/m^2^.

### Hypertension

Patients were considered to be suffering from hypertension if they were on antihypertensive medication at the time of participating in the study or the past medical history/record documented raised blood pressure (BP) at multiple occasions or the BP was recorded higher than average (>140/90 mmHg) on multiple separate occasions^19^.

### Laboratory tests

Fasting blood sugar (FBS), total cholesterol, and triglycerides (TG) were measured by using HP-CHEM250Y autoanalyzer. The cut-offs for abnormal levels were: TG ≥150 mg/dL, total cholesterol ≥200 mg/dL; while individuals with a random blood sugar of ≥200mg/dL were later confirmed by FBS, with an FBS ≥126 mg/dL considered diabetic^20^.

### Nass use

In this section, some questions were asked about nass use (status, frequency, duration). The information about nass use was obtained through face-to-face interviews. Respondents were classified as follows: current nass users reported using nass once or more times within the past 30 days, past nass users were defined as lifetime regular users not currently using nass. Non-users were defined as having not consumed nass in the their lifetime^21,22^.

### Statistical analysis

All analyses were performed with the SPSS 24.0 software package (SPSS, Armonk, NY: IBM Corp). Nass use rates are shown with 95% confidence intervals (CIs) for current nass users. Continuous and categorical data were compared using the t-test and χ^2^-test, respectively. Binary logistic regression was performed to identify the factors associated with nass consumption. A p<0.05 was considered significant.

## RESULTS

Participant characteristics are summarized in Table 1. A total of 387 patients were included in this study, of these, 189 were nass users and 198 were nonusers. The mean age and standard deviation was 63.9 (17.2) years for nass users and 42.3 (17.4) years for non-users. The male-to-female ratio among patients was 1.4, while that in a previous study was 0.6^23^. The overall prevalence of nass use was 48.8% (95% CI: 43.8–53.9%), with the Turkmen ethnic group having significantly higher prevalence than other ethnic groups (58.2%, 95% CI: 50.8–65.3% vs 41.8%, 95% CI: 34.7–49.2%). The majority of nass users (89.4%) were illiterate. The nass use was significantly higher among unemployed patients (27.0% vs 22.2%; p=0.001). Rural residence was associated with nass use (69.8% vs 32.8%; p<0.001). The employment rates were significantly lower among nass users than non-users (24.8% vs 39.8%; p=0.001). Rural residence was associated with nass use (69.8% vs 28.8%; p<0.001). There was a significant difference in the mean (SD) systolic pressure between patients who were nass users compared with non-users [129 (22) vs 136 (24); p=0.004]. The mean diastolic blood pressure was significantly lower among nass users than non-users [65.4 (12.7) vs 89.1 (16.3); p=0.013].

**Table 1:** Distribution of factors associated with nass consumption among the participants

	*Users N=189*	*Non-users N=198*	*P*
**Age**, mean (SD), years	63.9 (17.2)	42.3 (17.4)	<0.001
**Sex**, n (%)			0.663
Males	112 (59.2)	113 (57.0)	
Females	77 (40.7)	85 (42.9)	
**Level of education,** n (%)			<0.001
Illiterate	169 (89.4)	90 (45.4)	
Primary/private education	8 (4.2)	28 (14.1)	
Secondary	4 (2.1)	33 (16.6)	
High school or more	8 (4.2)	47 (23.7)	
**Marital Status,** n (%)			0.282
Single	34 (18.0)	25 (12.6)	
Married	146 (77.2)	160 (80.8)	
Others	9 (4.8)	13 (6.5)	
**Ethnicity**, n (%)			<0.001
Turkmen	110 (58.2)	56 (28.3)	
Other ethnic groups	79 (41.8)	142 (71.7)	
**Occupation,** n (%)			0.001
Employed	47 (24.9)	79 (39.8)	
Unemployed	14 (7.4)	24 (12.1)	
Housewife	51 (27.0)	44 (22.2)	
Farmers	77 (40.7)	51 (25.7)	
**Place of residence,** n (%)			<0.001
Rural	132 (69.8)	62 (31.3)	
Urban	57 (30.2)	136 (68.7)	
**History of family nass use, n (%)**	98 (51.8)	10 (5.0)	<0.001
**Measurements and tests**, mean (SD)			
Systolic blood pressure (mmHg)	129.3±22.0	136.0±23.9	0.004
Diastolic blood pressure (mmHg)	65.4±12.7	89.1±16.3	0.013
Body mass index (kg/m2)	23.4±3.1	23.5±3.9	0.835
Fasting blood sugar (mg/dL)	108.2±42.8	104.4±25.7	0.290
Total cholesterol (mg/dL)	174.1±30.4	173.1±32.7	0.744
Triglyceride (mg/dL)	159.5±38.9	160.3±38.4	0.836

Table 2 shows the results of a binary logistic regression model determining the relation between nass use and family history of nass use, level of education, rural residency, occupation status, ethnicity, and age.

**Table 2:** Odd ratio of risk factors for nass consumption among study participants

*Variables*	*N*	*Nass users*	*OR*	*(95% CI)*	*p*
**Family member using nass**					
No	279	91	1.0		
Yes	108	98	31.4	(12.5–78.5)	<0.001
**Education**					
High school or more	55	8	1.0		
Secondary	37	4	5.1	(1.7–15.4)	0.004
Primary/private education	36	8	5.0	(1.4–17.9)	0.014
Illiterate	259	169	8.9	(2.5–31.2)	0.001
**Occupation**					
Employed	126	47	1.0		
Housewife	95	51	1.0	(0.5–2.8)	0.017
Farmers	128	77	0.4	(0.2–0.8)	0.927
Unemployed	38	14	5.4	(1.4–21.4)	0.017
**Residence place**					
Urban residents	193	62	1.0		
Rural residents	194	132	2.0	(1.0–3.9)	0.044
**Age,** years					
≤24	36	6	1.0		
25–44	102	22	0.3	(0.1–1.7)	0.175
≥45	249	161	0.1	(0–0.4)	0.003
**Ethnicity**					
Other ethnic groups	221	79	1.0		
Turkmen ethnic group	166	110	1.8	(0.9–3.3)	0.074

Nass use was considered a dependent variable. Patients with family history of nass use were 31.4 times more likely to use nass (95% CI: 12.5–78.5). The odds of nass use were 8.9 higher among individuals with no education compared to those with high school or tertiary education (95% CI: 2.5– 78.5). The odds of nass use among rural residents were 2.0 times higher compared to urban residents (95% CI: 1.0–3.9), while the odds of the unemployed using nass were 5.4 times higher (95% CI: 1.4–21.4) than for those who were employed.

## DISCUSSION

To our knowledge, this is the first study to investigate the prevalence of nass use and its predictors among the Afghan population. SLT use has traditionally been part of the society and culture of the people of South and Southeast Asia^24^. Previous studies from Karachi showed that nass use played a strong role in the etiology of oral cancer in Pakistan^25,26^. The findings of our study showed that about half of the patients were using nass. Nass use was associated with the factors: family history of nass use, illiteracy, unemployment, and rural residency.

The present study showed that there was a high level of nass consumption among adult patients who visited an outpatient clinic in Andkhoy, Afghanistan. This is in line with a previous study conducted with adult patients who visited family medicine clinics in Karachi, Pakistan^27^. The reasons for the high rates of nass consumption might be that nass use is more acceptable among people in this region, it is inexpensive, easily available, and easier to use than cigarettes. There is also no law prohibiting nass sales, the sale of nass is not monitored in Afghanistan, and some smokers may also supplement their tobacco dependency habit by nass use. While there are antitobacco campaigns in Afghanistan, these mainly target cigarette smokers and messages against nass are often absent. It is also possible that due to the increasing taxation on cigarettes by the government of Afghanistan, the high cigarette prices might be a reason for some smokers to consider nass use as an alternative to smoking. Another reason for the high rate of nass use could be industry marketing: in recent years, nass is being sold in more attractive and colorful sachets and has also been made more flavorsome.

Afghanistan has ratified the WHO Framework Convention on Tobacco Control (FCTC) and on 2 Feb 2015 the law on tobacco control was enacted. The national law, which is supported by Islamic Shari’ah Law, includes banning the production, promotion, and use of cigarettes, water-pipe, hookah, snuff, and other tobacco products. It aims to help tobacco users to quit tobacco use, warn about the dangers of tobacco, and raise taxes on tobacco. However, the laws are not implemented exactly as they should be in Afghanistan^28,29^.

In our study, the use of nass was higher among the Turkmen ethnic group compared to other ethnic groups. One possible explanation might be that use of nass has been socially and culturally acceptable for Turkmen people for generations. For this reason, Turkmen nass consumers believe that nass use is part of their tradition.

Use of nass was higher among those participants who had at least one family member that used nass. A similar finding has been reported in another study among adult patients who visited family practice clinics in Pakistan^30^. It is believed that the role of family members in the initiation of nass use is more significant than that of society, peers, or other factors^31^. Moreover, when the habit is initiated before 18 years of age, the first lesson of smoking and tobacco consumption is often acquired at home from family members^32^. The most likely reason might be easy access to nass at home.

In our study, nass consumption is inversely related to the level of education. Similar findings have been reported from a previous study^33^. This might be due to the lack of awareness about the harmful effects of SLT and also that the death rate and overall risk taking behavior tends to be higher among the less educated^34^. It is likely that lack of awareness about the harmful effects of nass will lead the less educated in Afghanistan to situations predisposing them to nass use. It is also possible that the anti-tobacco educational campaigns initiated by the Afghan Ministry of Public Heath are limited in their effectiveness.

In our study, nass use was higher among unemployed people. This association with poverty has been seen in a previous study^35^. It is believed that tobacco may be used as a coping mechanism associated with the reduction of stress^36^.

Our finding is that nass use is prevalent among rural residents, which is similar to results of previous surveys in Bangladesh^37^. This could be due to lack of awareness among rural residents. Also, for socioeconomically disadvantaged Afghan people in rural areas, nass is locally manufactured, inexpensive, and easily available.

### Limitations

This study has some limitations. The sample size consisted of a single-center data source. The single-center nature of this study may affect its generalizability to the entire population of Afghanistan. Furthermore, the patients were selected on a convenience basis, which could have led to a potential source of bias. In addition, the information about nass use was obtained through face-to-face interviews administered through a structured questionnaire. Due to social reasons associated with tobacco and nass use, people may under-report their nass use. Therefore, self-reported nass use among participants is only reliable when tested by a serum cotinine level as indicator of nicotine metabolism among people who use both smoking and smokeless tobacco^38^. In addition, we determined the prevalence of nass use in a clinical setting. However, conducting this study among the wider population would be more effective in truly representing the prevalence of nass use. Furthermore, this study is based on a non-probability sample, and therefore cannot be generalized beyond the study population. Also, we did not perform a statistical test like Cronbach’s alpha for the questionnaire and there was only face validity for the questionnaire.

Finally, the cross-sectional nature of the study makes it difficult to interpret the correlation between nass use and its associated factors.

On the other hand, our data can be valuable for the accumulation of additional data regarding nass use, as our study was the first on prevalence and risk factors of nass use among adult patients attending an outpatient clinic in Afghanistan.

## CONCLUSIONS

This study showed that about half of the participants who were adult patients attending an outpatient clinic in Afghanistan were nass users. Nass use was more common among the Turkmen ethnic group. Factors associated with nass use were family history of nass use, illiteracy, unemployment, and rural residency. Our findings greatly reinforce the need to develop strategies for the control of nass consumption in Afghanistan.
