# Supplementary material for: Nass use and associated factors among outpatients in northern Afghanistan: A cross-sectional study in Andkhoy City
Source: Tob Induc Dis. 2018 Aug 24;16:36. doi: 10.18332/tid/93574 (PMC6659476; doi:10.18332/tid/93574)
Supplement: Supplementary file 1 [file TID-16-36-s1.pdf]

Appendix

Questionnaire about nass use

EXPANDED: Tobacco Use		
**Question**	**Response**	**Code**
How old were you when you **stopped** smoking?	Age (years)<br>Don't Know 77 └─┴─┘ *If Known, go to T12*	T10
How **long ago** did you stop smoking?	Years ago └─┴─┘ *If Known, go to T12*	T11a
(RECORD ONLY 1, NOT ALL 3)	OR Months ago └─┴─┘ *If Known, go to T12*	T11b
Don't Know 77	OR Weeks ago └─┴─┘	T11c
Do you **currently use** any **smokeless tobacco** products [such as *snuff, chewing tobacco, betel*]? *(USE SHOWCARD)*	Yes 1<br>No 2 *If No, go to T15*	T12
Do you **currently use smokeless tobacco** products **daily?**	Yes 1<br>No 2 *If No, go to T14aw*	T13
On average, how many **times a day/week** do you use ….<br>*(IF LESS THAN DAILY, RECORD WEEKLY)*<br>*(RECORD FOR EACH TYPE, USE SHOWCARD)*<br>*Don't Know 7777*	DAILY↓ WEEKLY↓	
	Snuff, by mouth └─┴─┴─┴─┘ └─┴─┴─┴─┘	T14a/<br>T14aw
	Snuff, by nose └─┴─┴─┴─┘ └─┴─┴─┴─┘	T14b/<br>T14bw
	Chewing tobacco └─┴─┴─┴─┘ └─┴─┴─┴─┘	T14c/<br>T14cw
	Betel, quid └─┴─┴─┴─┘ └─┴─┴─┴─┘	T14d/<br>T14dw
	Other └─┴─┴─┴─┘ └─┴─┴─┴─┘<br>*If Other, go to T14other, if T13=No, go to T16, else go to T17*	T14e/<br>T14ew
	Other (please specify): └─┴─┴─┴─┴─┴─┘<br>*If T13=No, go to T16, else go to T17*	T14other/<br>T14otherw
In the **past**, did you **ever use** smokeless tobacco products [such as *snuff, chewing tobacco, or betel*]?	Yes 1<br>No 2 *If No, go to T17*	T15

In the **past**, did you **ever use** smokeless tobacco products [such as *snuff, chewing tobacco, or betel*] **daily**?	Yes 1<br>No 2	T16
During the past 30 days, did someone smoke **in your home**?	Yes 1<br>No 2	T17
During the past 30 days, did someone smoke in closed areas **in your workplace** (in the building, in a work area, or a specific office)?	Yes 1<br>No 2<br>Don't work in a closed area 3	T18
